# Reduced SOD2 expression is associated with mortality of hepatocellular carcinoma patients in a mutant p53-dependent manner

**DOI:** 10.18632/aging.100967

**Published:** 2016-05-23

**Authors:** Ren Wang, Chen Yin, Xiao-Xing Li, Xian-Zi Yang, Yang Yang, Mei-Yin Zhang, Hui-Yun Wang, X.F. Steven Zheng

**Affiliations:** ^1^ State Key Laboratory of Oncology in South China, Collaborative Innovation Center for Cancer Medicine, Sun Yat-Sen University Cancer Center, Guangzhou, 510060, PR China; ^2^ Rutgers Cancer Institute of New Jersey, and Department of Pharmacology, Robert Wood Johnson Medical School, Rutgers, The State University of New Jersey, New Brunswick, NJ 08903, USA

**Keywords:** Superoxide Dismutase 2 (SOD2), Reactive Oxygen Species (ROS), liver, Hepatocellular Carcinoma (HCC), cancer

## Abstract

The development and progression of hepatocellular carcinoma (HCC) is accompanied with persistent oxidative stress, but the molecular basis is not well defined. Superoxide dismutase 2 (SOD2) is an important mitochondrial antioxidant and a key aging factor. Here we investigated the expression and clinical significance of SOD2 in a large cohort of HBV-positive HCC tumors. Both SOD2 mRNA and protein are reduced in human primary HCCs compared with matching liver tissues. Consistently, the SOD2 DNA copy numbers are decreased in HCCs, providing a genetic basis for the decrease in SOD2 mRNA expression. Reduced SOD2 expression in HCCs is correlated with older age, larger tumor size, multiple tumor nodules and tumor emboli, and cancer recurrence. Moreover, low SOD2 expression is strongly associated with poor overall survival (OS) and recurrence-free survival (RFS). Univariate and multivariate Cox regression analyses indicates that SOD2 is an independent prognostic predictor for OS and RFS. Intriguingly, reduced SOD2 mRNA is strongly associated with poor survival in a separate cohort of HCC patients carrying mutant p53. Altogether, our results provide clinical evidence for the importance of SOD2 in tumor progression and mortality, and the close relationship of SOD2 and p53 in HCC.

## INTRODUCTION

Reactive oxygen species (ROS) are oxygen-containing free radicals and reactive molecules such as superoxide (O_2_^−^), hydroxyl (OH^−^) and hydrogen peroxide (H_2_O_2_) [1]. ROS is generated during mitochondrial respiration and in enzyme-catalyzed reactions [2]. Mitochondrial respiration is a main source of ROS due to production of superoxide anion from Complex I and III of the electron transport chain, estimated at 1-2% oxygen consumed by the cell [3]. Superoxide anion is further converted into other ROS species such as hydroxyl free radical and hydrogen peroxide. ROS also plays an important regulatory role in many cellular activities such as metabolism and signal transduction [4, 5]. For example, H_2_O_2_ regulates protein tyrosine and lipid phosphatases, thereby dictating the signal transduction strength by receptor tyrosine kinases [6, 7]. However, under pathological conditions, ROS can reach excessively high levels, which is a major cause of aging and aging-related diseases such as Alzheimer's disease and cancer [8-10].

Because of the importance of ROS in normal physiology and diseases, eukaryotic cells have developed a sophisticated antioxidant network to maintain redox homeostasis. The superoxide dismutases (SOD), catalases, thioredoxin and glutathione are key antioxidant enzymes that remove ROS from the cellular environment. SOD is a family of evolutionarily conserved enzymes that dismutate superoxide free radicals. In mammals, SOD family has three distinct members: SOD1 or the copper/zinc SOD, SOD2 or the manganese SOD and SOD3 or the extracellular SOD [11, 12]. SOD1 is the major type of SOD that is found in the cytoplasm, nucleus and mitochondrial inter-membrane space [13, 14]. SOD2 is only localized in the mitochondrial matrix [15] where it is associated with mtDNA and thought to prevent oxidization of mtDNA and mtDNA polymerase from being oxidized [16]. Mutations of SOD2 have been shown to alter lifespan in a variety of model organisms such as C. elegance and Drosophila [17, 18]. SOD2 level and activity may contribute to metabolic reprogramming in response to nutrient and stressful conditions [19]. Homozygous SOD2 knockout mice die shortly after birth [20], while heterozygous SOD1 knockout mice exhibit elevated mitochondrial oxidative stress and age-dependent decline of mitochondrial function [21].

Liver cancer is one of the most prevalent and deadly human malignancies [22, 23]. Hepatocellular Carcinoma (HCC), the predominant type of liver cancer, accounts for about 85% of total liver cancer cases [24]. Hepatic resection is the main curative treatment for early stage diseases. Unfortunately, patients after hepatectomy still have a high rate of distant metastasis and inhepatic relapse, with approximately 50% chance of 5-years recurrence. Therefore, it is important to identify molecular markers to predict disease recurrence and prognosis. Due to the important role of SOD1 in mitochondrial function and integrity, it is not surprisingly that SOD2 has been linked to tumorigenesis [11, 12]. SOD2 expression was found to be down in breast [19], esophageal [25], and pancreatic cancer [26], but up in ovarian [27], gastric [28] and colorectal cancer [29]. Thus the role of SOD2 appears to be tumor-suppressive or -promoting, dependent on tissue types and/or disease stages. Mitochondria play crucial roles in the normal physiological functions and pathogenesis of the liver [30]. Despite the importance of SOD2 in mitochondria, to date, SOD2 has not been studied in liver cancer. This study was aimed to determine the expression and clinical significance of SOD2 in HCC.

## RESULTS

### SOD2 expression is reduced in human HCC

**Figure 1:**
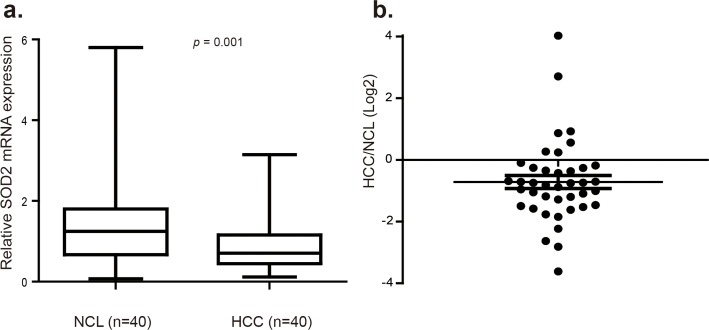
SOD2 mRNA expression is down-regulated in primary human HCC tissues (**a**) Relative SOD2 mRNA expression was detected by RT-qPCR in 40 paired primary human HCC tissues and adjacent non-cancerous liver (NCL) tissues. (**b**) Relative SOD2 mRNA expression level in individual tumors versus matching NCL tissues.

Altered SOD2 level has been reported in several cancer types, but the pattern is complex with down-regulation in some tumors but up-regulation in others. Thus far, SOD2 expression has not been investigated in HCC. Given the importance of SOD2 in mitochondrial integrity and liver biology, we investigated SOD2 expression in HCC. To this end, SOD2 mRNA level was analyzed in 40 pairs of human primary HCC and matching adjacent non-cancerous liver (NCL) tissues by RT-qPCR. SOD2 mRNA expression was found to be significantly reduced in 30 of 40 tumors, representing 75% of the HCC tissues compared with the NCL tissues (*p* = 0.001, Fig. 1a and 1b). In tumors with SOD2 down-regulation, SOD2 expression was reduced by as much as 12-fold, with the median decrease nearly 2-fold (Fig. 1b).

**Figure 2:**
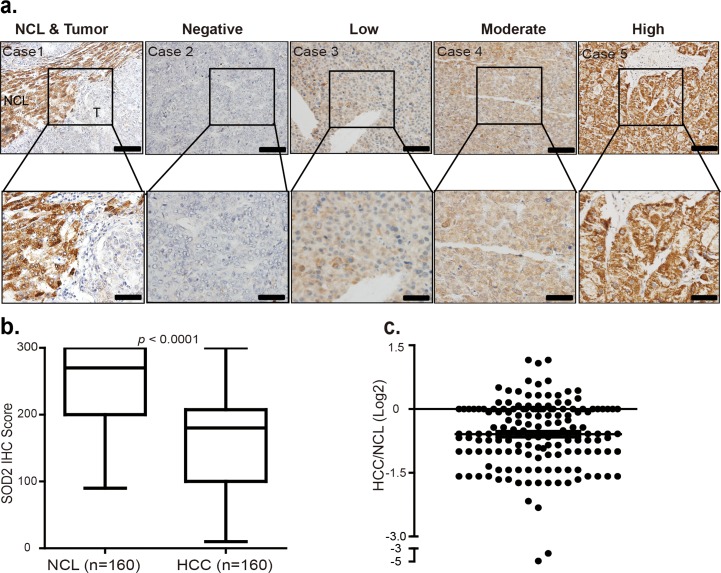
SOD2 protein level is decreased in primary human HCC tissues (**a**) Immunohistochemistry (IHC) staining of SOD2 in HCC tissues and adjacent non-cancerous liver tissues. Shown are representative images of negative, low, moderate and high SOD2 IHC staining. (**b**) Box plot graph of SOD2 IHC staining scores in HCC and matching NCL tissues. Data statistical analysis were performed by Sample-Paired t-test. (**c**) Scatter plot shows SOD2 staining level in individual tumors as a ratio of SOD2 staining in HCC tissues versus paired NCL tissues.

To verify this finding, we investigated SOD2 protein expression by immunohistochemistry (IHC) staining of a large cohort of 160 paraffin-fixed human primary HCC tumors and matching adjacent NCL tissues. Based on the study of genomic mRNA expression profiling in different mouse tissues [31], liver is one of the tissues where SOD2 is highly expressed in mice (Fig. S1). Consistently, SOD2 was found to be abundant as indicated by strong IHC staining in most of the NCL tissues (Fig. 2a). However, in tumor tissues, SOD2 protein expression showed considerably variations, ranging from negative, low, moderate to high IHC staining (Fig. 2a). Quantification of SOD2 staining IHC scores confirmed that SOD2 is indeed significantly decreased in HCC tissues as compared with their matched NCL tissues (p < 0.001, Fig. 2b). SOD2 protein expression was found to be largely reduced in 111 of 160 (69%) patients HCC tissues compared with the NCL tissues (*p* < 0.0001, Fig. 2b and 2c). In these 111 patients' HCC tissues, SOD2 expression was reduced by as much as 30-fold, with the median decrease 1.67-fold (Fig. 2c). Together, these results show that SOD2 expression is reduced at both mRNA and protein level in HCC.

### Mechanism of SOD2 down-regulation in HCC

**Figure 3:**
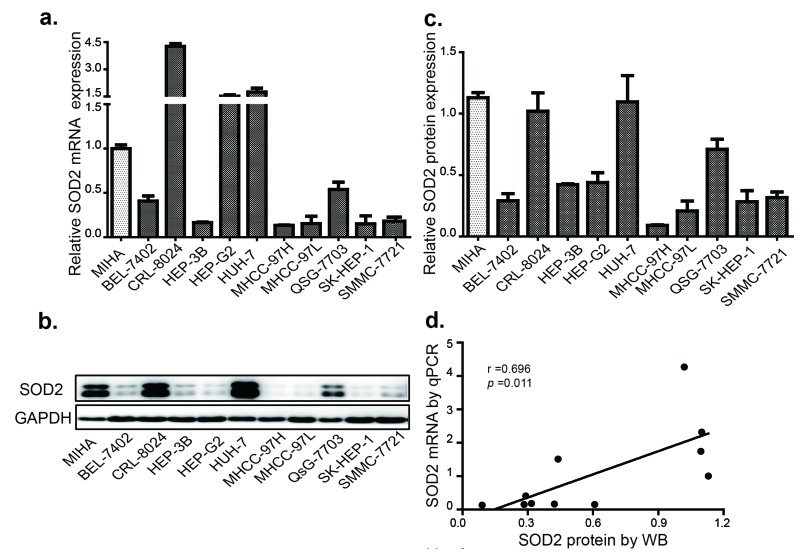
SOD2 expression is decreased in HCC and cell lines (**a**) The relative expression of SOD2 mRNA in HCC cell lines. (**b**) The SOD2 protein expression in HCC cell lines as determined by Western blot. (**c**) Quantification of the results from Fig. 3b. (**d**) Correlation of SOD2 mRNA and protein expression in HCC cell lines. Statistical analysis was conducted by Paired-Samples t-test.

**Figure 4:**
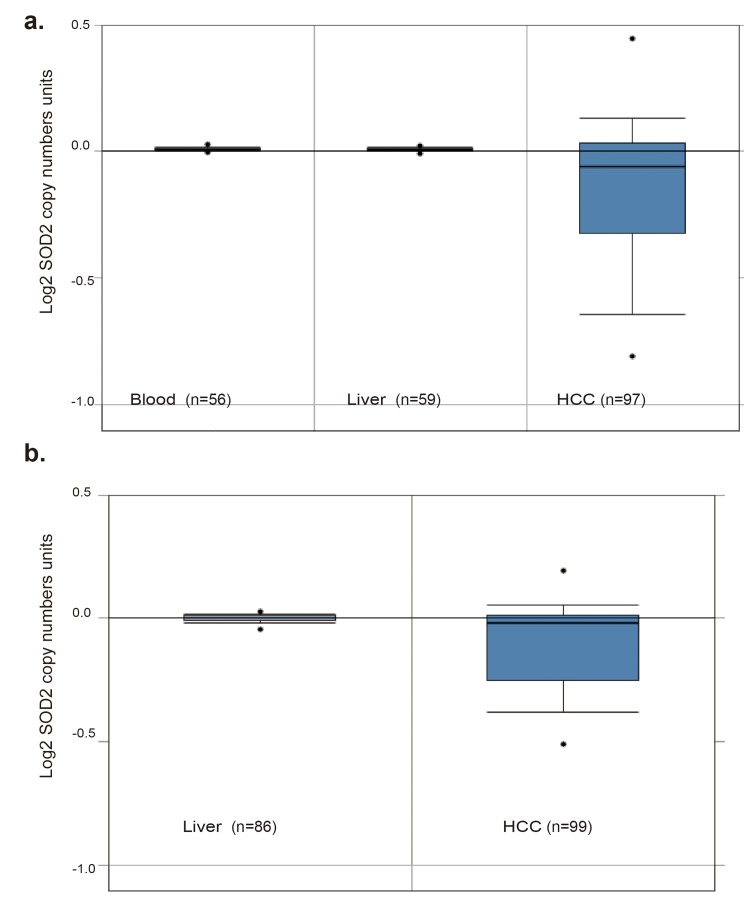
SOD2 DNA copy number is decreased in primary human HCC tissues (**a**) SOD2 DNA copy number inHCC tumors, and matching normal liver and blood samples obtained from the TCGA Cancer Genomic Database. (**b**) SOD2 DNA copy number in HCC tumors and matching normal liver samples of Guichard Liver downloaded from the OncoMine cancer genomic database.

To understand the relationship between SOD2 mRNA and protein expression in HCC, we analyzed a panel of 10 HCC cell lines and an immortalized human hepatocyte cell line by RT-qPCR and Western blotting. Compared with the immortalized hepatocyte cell line MIHA, SOD2 mRNA was found to be lower in 7 of the 10 HCC cell lines (Fig. 3a), and protein level was lower in 8 of 10 HCC cell lines (Fig. 3b and 3c). The mRNA and protein level are largely correlated with each other (Fig. 3d), suggesting that SOD2 mRNA abundance is the primary determinant of SOD2 expression. However, there are some exceptions. Specifically, although SOD2 mRNA in HepG2 cells was higher than MIHA cells, SOD2 protein level was actually lower in HepG2 cells. QSG-7703 showed decreased SOD2 mRNA but not protein compared with MIHA cells. Thus, translational and post-translational mechanisms are likely to be involved in these cases. To understand the mechanism for altered SOD2 mRNA expression, we analyzed SOD2 copy number changes in one cohort of 97 HCC, 59 normal liver and 57 blood samples from the TCGA cancer genomic database (http://cancergenome.nih.gov). There was a pronounced decrease in SOD2 copy number in HCC versus blood and normal liver samples (Fig. 4a). Essentially the same phenomenon was observed with another cohort of 99 HCC and 86 normal liver samples obtained from the Oncomine genomic database (Fig. 4b) [32]. These observations indicate that loss of SOD2 locus is a mechanism for the decrease in SOD2 mRNA expression in HCC.

### Loss of SOD2 expression is associated with advanced age and cancer progression in HCC patients

Frequent down-regulation of SOD2 suggests that it plays an important role in HCC pathogenesis. We therefore investigated the relationship between SOD2 expression and clinicopathological features of HCC. Based on the IHC scores, we divided HCC patients into SOD2 high-expression and low-expression subgroups using the median IHC score of 180 as the cutoff value. We then analyzed the correlation between SOD2 expression and 15 widely recognized clinicopathologic parameters in the cohort of 160 HCC specimens (Table 1). Consistent with the established role of SOD2 in aging, chi-square analysis shows that there is a statistically significant correlation between low expression of SOD2 and older patients (≥ 50 y, *p* = 0.007). Moreover, low expression of SOD2 is associated with multiple primary tumors (*p* = 0.012), tumor embolus (*p* = 0.016), tumor recurrence (*p* = 0.039), and advanced tumor stages as classified by the Primary Tumor, Lymph Nodes and Metastasis (TNM) (*p* = 0.006), and Barcelona clinic liver cancer (BCLC) (*p* = 0.001) staging systems. These observations suggest that low SOD2 expression is involved in the progression of HCC.

**Table 1:** Correlation between the clinicopathological features and SOD2 protein expression

Features		SOD2 protein expression		
	No. of cases	Low	High	*p* value
**Age**				
≥ 50 years	83	53 (63.9%)	30 (36.1%)	**0.007**
< 50 years	77	32 (41.6%)	45 (59.4%)	
**Gender**				
Female	18	12 (66.7%)	6 (33.3%)	0.316
Male	142	73 (51.4%)	69 (48.6%)	
**HBV DNA copy**				
≤ 1000	58	31 (53.4%)	27 (46.6%)	0.516
> 1000	102	54 (52.9%)	48 (47.1%)	
**Serum HBsAg**				
Negative	13	6 (46.2%)	7 (53.8%)	0.414
Positive	145	78 (53.8%)	67 (46.2%)	
**Serum AFP (ng/ml)**				
< 400	71	32 (45.1%)	39 (54.9%)	0.112
≥ 400	92	53 (57.6%)	39 (43.4%)	
**Liver Cirrhosis**				
NO	63	37 (56.9%)	26 (43.1%)	0.262
YES	97	48 (49.5%)	49 (50.5%)	
**Tumor Differentiation**				
I-II	92	48 (52.2%)	44 (47.8%)	0.873
III-IV	68	37 (54.4%)	31 (45.6%)	
**Tumor Size (cm)**				
< 5	59	31 (52.5%)	28 (47.5%)	0.520
≥5	101	54 (53.5%)	47 (46.5%)	
**Tumor Number**				
1	117	55 (47.0%)	62 (53.0%)	**0.012**
> 1	43	30 (69.8%)	13 (30.2%)	
**Tumor Capsule**				
Complete	128	68 (53.1%)	60 (46.9%)	0.579
Incomplete	32	17 (53.1%)	15 (46.9%)	
**Tumor Embolus**				
No	135	66 (48.9%)	64 (51.1%)	**0.016**
Yes	25	19 (76.0%)	6 (24.0%)	
**Postoperative Metastasis**				
No	151	79 (52.3%)	72 (47.7%)	0.503
Yes	9	6 (66.7%)	3 (33.3%)	
**Recurrence**				
No	97	44 (45.4%)	53 (54.6%)	**0.039**
Early (<24 months)	39	24 (65.1%)	15 (34.9%)	
Late (≥24 months)	24	17 (45.4%)	7 (54.6%)	
**TNM Stage**				
I-II	121	57 (47.1%)	64 (52.9%)	**0.006**
III	39	28 (71.8%)	11 (28.2%)	
**BCLC Stage**				
0-A	102	44 (43.1%)	58 (56.9%)	**0.001**
B-C	58	41 (42.5%)	17 (57.5%)	

### Reduced SOD2 expression is significantly correlated with poor prognosis of HCC patients

**Figure 5:**
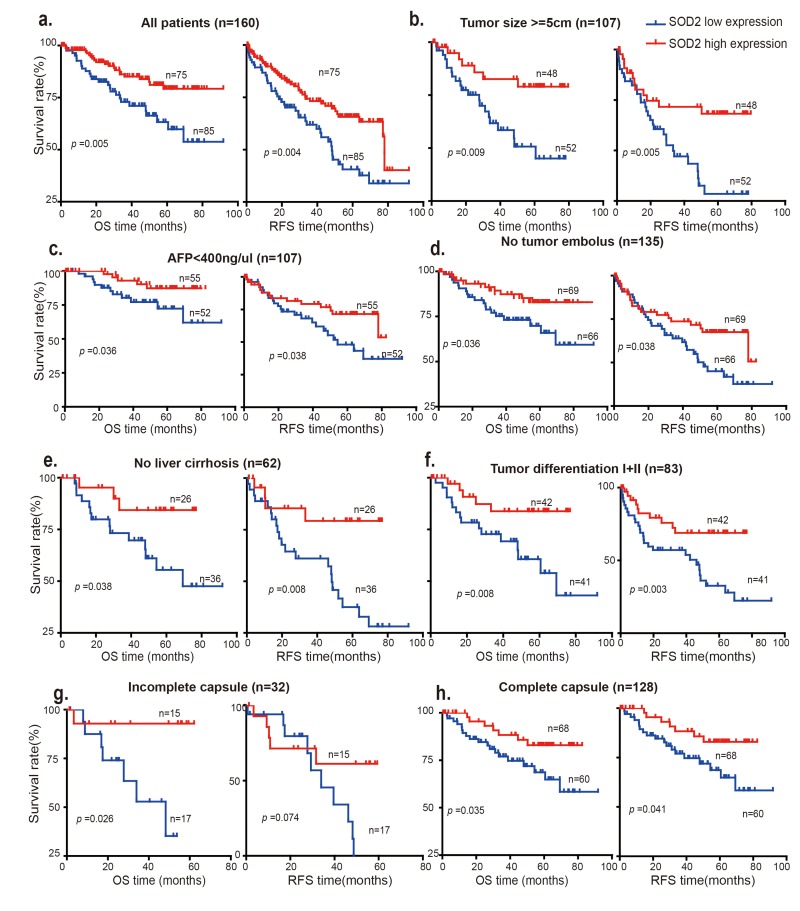
Low SOD2 protein expression is correlated with overall survival (OS) and relapse-free survival (RFS) The OS and RFS of 160 HCC patients with SOD2 high expression and low expression are analyzed in (**a**) all patients, (**b**) patients with tumor size >5cm, (**c**) patients with AFP <400 ng/μl), (**d**) patients without tumor embolus, (**e**) patients with no liver cirrhosis, (**f**) patients with tumor differentiation I+II, (**g**) patients with incomplete capsule, (**h**) patients with complete capsule.

Because loss of SOD2 is associated with HCC progression, we investigated that whether differential SOD2 expression is linked to the prognosis of HCC patients. To this end, Kaplan-Meier and the log-rank test analysis were used to evaluate the survival of these HCC patients. As shown in Fig. 5a, patients with low SOD2 expression had statistically significant, shorter overall survival (OS) and relapse-free survival (RFS) than those with high SOD1 expression (*p* = 0.005 and *p* = 0.004, respectively). The postoperative median OS and RFS times of the entire cohort of 160 HCC patients were 40.87 months and 30.08 months respectively, and for the 75 patients with high SOD2 expression were 50.27 and 46.17 months, respectively. In contrast, the postoperative median OS and RFS times for the 85 cases with low SOD2 expression were 34.1 and 29.03 months, respectively. Furthermore, low SOD2 protein expression correlates with worse prognosis for patients with large tumors (tumor size > 5cm) (Fig. 5b).

Interestingly, low SOD2 expression is also associated with shorter OS and RFS in subgroups of patients characteristic of less advanced disease, including low AFP concentration (< 400 ng/μl) (Fig. 5c), no tumor embolus (Fig. 5d), no liver cirrhosis (Fig. 5e), high differentiation (Grade I+II) (Fig. 5f) and complete tumor capsule (*p* = 0.035) subgroup, suggesting that SOD2 is involved in the development and progression of HCC during early disease stages. Low SOD2 expression also appears to be associated with poorer prognosis in more advanced disease stages (Fig. S2). However, surgical HCC samples were usually obtained in relatively early disease stages, which limits the sample size and thus statistical significance. Further study will be necessary to elucidate the clinical significance of SOD2 expression in advanced HCC.

### SOD2 is an independent prognostic predictor for HCC patients

To investigate the prognostic value of SOD2 protein expression, univariate analysis of SOD2 protein level and clinicopathological features was carried out using the Cox proportional hazard model. The result shows that low SOD2 protein expression (*p* = 0.005), higher serum AFP (*p* = 0.002), larger tumor size (*p* = 0.010), multiple tumors (*p* < 0.001), tumor embolus (*p* = 0.004) advanced TNM (*p* = 0.001) and BCLC stage (*p* < 0.001) are predictors for poor OS of HCC patients (Table 2). On the other hand, low SOD2, higher Serum AFP, tumor embolus, multiple tumors, advanced TNM, BCLC, and postoperative metastasis were statistically significantly correlated with shorter RFS in HCC patients (Table 2). To determine if SOD2 low expression can be an independent prognostic factor, multivariate Cox regression was further performed on those variables significantly associated with poor survival of HCC patients. The results demonstrate that low SOD2 protein expression is indeed an independent predictor for poor OS (HR = 2.310, 95% CI = 1.031-5.178, *p* = 0.042) and RFS (HR = 1.809, 95% CI = 1.037-3.156, *p* = 0.037) in patients with HCC (Table 2), suggesting that SOD2 protein is a useful prognostic biomarker for HCC patients.

**Table 2:** Univariate and multivariate Cox regression analysis of SOD2 associated with survival and recurrence in patients with HCC

	Univariate analysis			Multivariate analysis		
Variables	HR	95% CI	*p* value	HR	95% CI	*p* value
**Overall Survival**						
SOD2 Expression (low vs. high)	2.036	1.024-4.427	**0.038**	1.774	1.167-3.570	**0.042**
Gender (Male vs. Female)	0.643	0.269-1.539	0.321			
Age (≥ 50y vs. < 50y)	0.817	0.431-1.551	0.537			
HBV DNA copy (≥ 1000 vs. < 1000)	0.924	0.473-1.808	0.818			
Serum HBsAg (positive vs. negative)	0.720	0.255-2.030	0.534			
Serum AFP (≥ 400 vs. < 400 ng/ml)	2.556	1.348-4.845	**0.004**	2.328	1.097-4.943	**0.016**
Liver Cirrhosis (yes vs. no)	0.863	0.455-1.636	0.651			
Tumor Differentiation (I—II vs. III—IV)	0.799	0.413-1.546	0.506			
Tumor Size (≥ 5 cm vs. < 5 cm)	2.161	1.049-4.364	**0.037**			
Tumor Number (> 1 vs. 1)	2.530	1.316-4.861	**0.005**	2.144	1.101-4.175	**0.025**
Tumor Embolus (yes vs. no)	2.653	1.185-5.940	**0.018**			
Tumor Capsule (yes vs. no)	1.301	0.592-2.858	0.513			
TNM Stage (I-II vs. III)	3.276	1.703-6.303	**< 0.001**			
BCLC Stage (0-A vs. B-C)	3.701	1.919-7.139	**< 0.001**			
Postoperative Metastasis (yes vs. no)	1.930	0.683-5.452	0.214			
**RECURRENCE-FREE SURVIVAL**						
SOD2 Expression (low vs. high)	2.429	1.200-4.919	**0.017**	1.704	1.003-2.894	**0.029**
Gender (Male vs. Female)	0.860	0.409-1.807	0.690			
Age (≥ 50y vs. < 50y)	1.429	0.749-2.727	0.279			
HBV DNA copy (≥ 1000 vs. < 1000)	0.510	0.241-1.078	0.078			
Serum HBsAg (positive vs. negative)	1.344	0.487-3.705	0.568			
Serum AFP (≥ 400 vs. < 400 ng/ml)	1.911	1.147-3.184	**0.013**			
Tumor Differentiation (I-II vs. III-IV)	0.271	0.119-0.618	**0.002**	0.240	0.104-0.554	**0.008**
Tumor Size (≥ 5 cm vs. < 5 cm)	1.496	0.762-2.939	0.242			
Tumor Number (> 1 vs. 1)	3.055	1.588-5.877	**0.001**	2.737	1.411-5.309	**0.001**
Tumor Embolus (yes vs. no)	1.015	0.354-2.905	0.979			
Tumor Capsule (yes vs. no)	1.090	0.476-2.498	0.838			
TNM Stage (I-II vs. III)	2.745	1392-5.411	**0.004**			
BCLC Stage (0-A vs. B-C)	2.605	1.358-4.998	**0.004**			
Postoperative Metastasis (yes vs. no)	2.784	1.081-7.170	**0.032**	2.887	1.104-7.547	**0.031**

### Increased mortality of HCC patients with low SOD2 expression is p53-dependent

**Figure 6:**
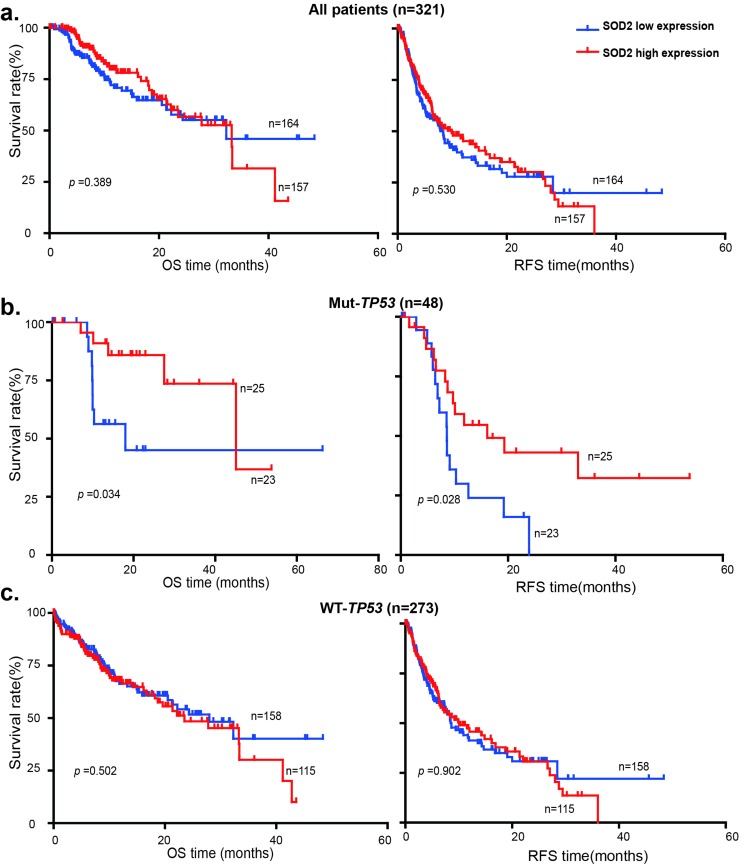
Low SOD2 mRNA expression is associated with poor survival in HCC patients with p53 mutations OS and RFS of 321 HCC patients with high SOD2 mRNA expression and low SOD2 mRNA expression in (**a**) all patients, (**b**) patients with mutant *TP53*, (**c**) patients with wild type *TP53*.

The tumor-suppressor p53 plays an important role in oxidative stress response. It has been shown that SOD2 is a p53 target gene at the transcriptional level: p53 normally binds to SOD2 promoter and represses SOD2 transcription [33], but activates SOD2 expression during oxidative stress [34]. Because *TP53* is frequently mutated in liver cancer, we investigated the relationship between *TP53* mutations and SOD2 expression in the survival of HCC patients using genomic and clinical data from cBioPortal for Cancer Genomics (http://www.cbioportal.org). HCC patients were divided into the SOD2 high expression and low expression subgroups using the median SOD2 mRNA value of 12645.314 as the cutoff. Kaplan-Meier and the log-rank test analysis showed that differential SOD2 mRNA expression is not statistically significantly correlated with patient survival in this cohort of HCC patients (Fig. 6a). We therefore further divided HCC patients into wild type and mutant *TP53* subgroups. Strikingly, low SOD2 mRNA expression is associated with much shorter OS (*p* < 0.034) and RFS (*p* < 0.028) in HCC patients carrying the mutant *TP53,* but not the wild type *TP53* (Fig. 6b). These results suggest that low SOD2 expression alters the mortality of this HCC patient population in a p53-dependent manner.

## DISCUSSION

In this study, we demonstrate that the SOD2 mRNA expression, protein expression and DNA copy number are significantly decreased in HCC tissues compared with the matched NCL tissues. Consistently, mRNA and protein expression of SOD2 in HCC cell lines is also markedly down-regulated, indicating that down-regulation of SOD2 is a common feature of HCC. Compared with matching liver and blood samples, there is also a pronounced loss of SOD2 copy number in the liver cancer tissues. Thus deletion of the SOD2 DNA locus is likely to be responsible for the reduced SOD2 transcription in HCC. The decrease in SOD2 mRNA is generally well correlated with reduced SOD2 protein expression. However, in HepG2 cells, SOD2 protein level is low despite of high SOD2 transcripts, indicating that post-transcriptional mechanism such as ubiquitin-mediated degradation [35] is involved.

Oxidative stress due to elevated ROS level, especially in the mitochondria, has been shown to play an important role in hepatocarcinogenesis [36-38]. As an important mitochondrial antioxidant enzyme, SOD2 has been shown to play a key role in mitochondrial function and integrity. Not surprisingly, SOD2 is increasingly recognized for its role in tumorigenesis. However, the expression of SOD2 in malignancies of different tissue origin varies significantly, from down-regulation in esophageal, pancreatic and breast cancer, to elevated level in ovarian, gastric and colorectal cancer. These observations indicate that the role of SOD2 is highly complex, which may act as a tumor suppressor or an oncogene, dependent on tumor tissue origin and/or disease stages. Homozygous SOD2 knockout mice is embryonic lethal [20], making it difficult to study the role of SOD2 deficiency in tumorigenesis in mice. SOD2^+/−^Gpx1^−/−^mice has increased incidents of tumorigenesis, but the tumors are primarily lymphoma and to a lesser degree lung adenocarcinoma [39]. The availability of large cohorts of primary human HCC tissue samples and genomic data provide extraordinary opportunity to understand the potential role of SOD2 expression in hepatocarcinogenesis. In this study, we show that SOD2 expression is commonly reduced in HCC, suggesting that it primarily has a tumor suppressive function in liver tumorigenesis.

It has been shown that SOD2^+/−^Gpx1^−/−^ mice develop lymphoma and lung adenocarcinomas in advanced age [39]. In agreement with SOD2 as a key aging factor, we found that reduced SOD2 protein expression in HCC is significantly associated with Chinese HCC patients in the older age group (> 50 year). Moreover, down-regulation of SOD2 protein expression is correlated with multiple tumors, tumor embolus, cancer recurrence, and more advanced tumor stages as defined by the TNM and BCLC staging systems. Kaplan-Meier analysis indicates the HCC patients with low SOD2 expression have a worse OS and RFS. Multivariate Cox regression analysis further shows that SOD2 protein level is an independent predictor for prognosis. These clinical data suggest that SOD2 has a tumor suppressive function in HCC tumorigenesis and progression and that SOD2 protein expression is a potentially useful prognostic biomarker in this patient population.

p53 is an important aging factor that controls cellular senescence [40, 41]. p53 regulates mitochondrial functions [42, 43] and redox homeostasis in part through the control of SOD2 expression [33, 34]. p53 is an important tumor suppressor in human cancer and *TP53* is estimated to be mutated in 30-50% of human liver cancer according to the cBioPortal (http://www.cbioportal.org). We have analyzed of the genomic data available for a total of 312 HCC patients in the TCGA cancer genomic database. Differential SOD2 mRNA expression was found to be not statistically significant correlated with survival, which is in contrast to the data for the cohort of Chinese HCC patients that shows low SOD2 protein level is associated with higher mortality rate. A possible explanation for the difference is etiology of HCC in the two different cohorts. While virtually all of the Chinese patients are HBV-positive, the TCGA patients are largely HBV-negative. The different etiology may explain the influence on mortality by SOD2. Another key difference is that the two studies used SOD2 protein and mRNA expression, respectively, as measuring metrics. Further comprehensive comparative study is necessary to fully understand the role of SOD2 in the disease development, progression and prognosis of HCC patients with distinct etiology. In the TCGA data, HCC patients with SOD2 low mRNA expression is significantly associated with much shorter OS (*p* < 0.034) and RFS (*p* < 0.028) in HCCs with mutant *TP53* than those with wild type *TP53*. Further study the relationship between p53 and SOD2 could lead to insights into the pathogenesis of liver cancer and may lead to new approaches to HCC prevention and therapy.

## MATERIALS AND METHODS

### Clinical specimens and cell lines

Formalin-fied paraffin-embedded (FFPE) HCC and matchingnon-cancerous liver (NCL)tissues from 160 patients with primary HCC, who underwent curative hepatectomy from January 2004 to August 2012, were collected from the Department of pathology of the Sun Yat-sen University Cancer Center, Guangzhou, China. The detailed clinicopathological features of patients are summarized in Table 1 and Table S1. The criteria of the 7th Edition tumor-node-metastasis (TNM) classification of the American Joint Committee on Cancer Staging [44] and the Barcelona Clinic Liver Cancer (BCLC) staging system [45] were used to determine the clinical stages. The cases were followed up regularly since the date of primary liver resection and the median follow-up time was 40.7 months. Overall survival (OS) was defined as the interval elapsed between the date of primary liver resection and the date of HCC-associated death or last follow-up. Recurrence-free survival (RFS) was calculated from the date of primary liver resection to the date of HCC-associated death, relapse, metastasis, or last follow-up. In addition, another 40 pairs of frozen HCC and matched NCL tissues were used for mRNA expression analysis. All the paired tissues were confirmed histologically by two pathologists of this cancer center. Written informed consent was obtained from all patients for this research. This study was approved by the Institutional Review Board and Human Ethics Committee of Sun Yat-Sen University Cancer Center prior to the study.

Human HCC cell line BEL-7402 is maintained in RPMI-1640 medium (Gibco, Carlsbad, CA) supplemented with 10% FBS. Human HCC cell lines CRL-8024, HEP-3B, HEP-G2, HUH-7, MHCC-97H, MHCC-97L, QSG-7703, SK-HEP-1 and SMMC-7721, and the immortalized human hepatocyte cell line MIHA, were cultured in DMEM (Life Technologies, Inc.) with supplemental 10% FBS. All cell lines were incubated at 37°C with 5% CO_2_.

### RNA extraction and RT-qPCR

Total RNA was extracted from the aforementioned cell lines and snap-frozen HCC/NCL tissues using the TRIzol reagent (Invitrogen, 15596-018, USA) according to the manufacturer's instruction. Two-step RT-qPCR was performed to assess the mRNA level of SOD2 as previously described [46]. First strand cDNA was synthesized by GoScript™ reverse transcriptas and random primers (Promega, A5001, Madison, WI, USA). cDNA was quantified in triplicate by GoTaq® qPCR Master Mix (Promega, A6001, Madison, WI, USA) on Roche LightCycler480(Roche, Basel, Switzerland). GAPDH was used as an internal control. Relative SOD2 mRNA expression was presented by 2−ΔΔCT method. Paired primer sequences used for SOD2 are: 5′-GACAAACCTCAGCCCTAACG-3′ (forward) and 5′-GAAACCAAGCCAACCCCAAC-3′ (reverse); for GAPDH: 5′-TGCACCACCAACTGCTTAGC-3′ (forward) and 5′-GGCATGGACTGTGGTCATGAG-3′ (reverse).

### Western blotting

Total proteins were extracted from the above-mentioned cell lines and fresh tissues by using a whole cell lysis kit (Keygen, KGP2100, China). The Western blot procedure is as previously described [47]. Briefly, 30 μg of total protein from each sample was separated in 12% SDS-PAGE gel and transferred to PVDF membrane (Millipore, Billerica, MA). After blocking in PBS buffer containing 6% nonfat milk at room temperature for 1 hr, the membrane was incubated with rabbit anti-SOD2 (1:5000, Cat No. ab13533, Abcam, USA) and anti-GAPDH (1:5000, Cat No. 5174, Cell Signaling Technology, USA) primary antibodies at 4°C overnight. After washing with TBS buffer containing 0.1% Triton X-100 for three times, the membrane was then probed with anti-rabbit HRP-conjugated secondary antibodies (dilution for 1:5000, Jackson Immunoresearch Inc, PA, USA) and detected by using the enhanced chemiluminescence substrates (PerkinElmer, MA, USA). GAPDH was used as a loading control. The intensity of immunoblot signals was determined using the Bio-Rad software Quantity One (Bio-Rad Laboratories Inc., Hercules, California, USA).

### Immunohistochemistry

Immunohistochemistry (IHC) was performed on 4 μM thick sections that were cut from the FFPE tissue blocks. The sections first were dried in an oven at 60°C for 2 hr, deparaffinized three times with xylene for 10 min each, rehydrated in a graded series of decreased alcohol solutions, washed three times with PBS for 10 min to remove the residual alcohol, immersed in 0.3% H_2_O_2_ in methanol to block endogenous peroxidase activity, washed three times with PBS for 10 min each to remove the residual H_2_O_2_ and methanol, boiled in 0.01 M sodium citrate buffer (pH 6.0) using a microwave oven for 15 min at low heat to retrieve the antigens, and cooled to room temperature by running water. After incubating in 10% FBS for 30 min to prevent nonspecific staining, the sections were incubated with rabbit anti-SOD2 (1:400, Cat No 13533, Abcam, USA) antibodies at 4°C overnight. In the second day, the tissue sections were rinsed three times with PBST (0.1% Triton-X100 in PBS), labeled with secondary antibody conjugated with horseradish peroxidase (Dako Corp., Carpinteria, CA) for 30 min at 37°C, washed three times with PBS for 10 min each to remove the remaining secondary antibodies, developed with 3, 3′-diaminoben-benzidine (Dako Corp., Carpinteria, CA), counterstained with hematoxylin (Sigma), dehydrated in increased concentration of alcohol, and dried on the air and mounted with neutral resins. All tissue slides were evaluated using the semi-quantitative immunostaining score (ISS) method by two pathologists who were blind to all clinical and biological variables. The score of immunostaining was defined as 0 – 3 (0, negative; 1, weak; 2, moderate; 3, strong). The percentage of positive immunostaining was scored as 0 to 100%, yielding an overall score ranging from 0 to 300 points. The median score was used as a cutoff for classification of patients into high and low expression groups.

### Statistical analysis

The SPSS software package for windows (version 17.0; Chicago, IL, USA) was used for all statistical analysis. Paired Student's t test was used to analyze the difference of mRNA and protein expression levels between the paired HCC and NCL tissues. The chi-square test was performed to determine the correlation of SOD2 expression with the patients' clinicopathological features. The Kaplan-Meier method and log-rank test were used to analyze survival of HCC patients. To determine if SOD2 is an independent factor of survival and recurrence, univariate and multivariate survival analyses were performed using the Cox proportional hazards regression model. The two-sided *p* value less than 0.05 was defined as statistically significant for all statistical analyses.

### Analysis of TCGA and Oncomine genomic data

We downloaded and analyzed SOD2 mRNA expression (RNA Seq V2 RSEM) data, the p53 mutant data and the clinical data of the Hepatocellular Carcinoma (TCGA, provisional) from the website cBioPortal (http://www.cbioportal.org/data_sets.jsp) [48, 49]. The SOD2 copy number data of TCGA (http://cancergenome.nih.gov/) and Guichard Liver [32] were downloaded and analyzed from the OncoMine cancer genomic database (https://www.oncomine.org/).

## SUPPLEMENTARY DATA FIGURES AND TABLE


