# Supplementary material for: Reduced SOD2 expression is associated with mortality of hepatocellular carcinoma patients in a mutant p53-dependent manner
Source: Aging (Albany NY). 2016 May 23;8(6):1184–97. doi: 10.18632/aging.100967 (PMC4931826; doi:10.18632/aging.100967)
Supplement: Supplementary file 1 [file aging-08-1184-s001.pdf]

SUPPLEMENTARY DATA

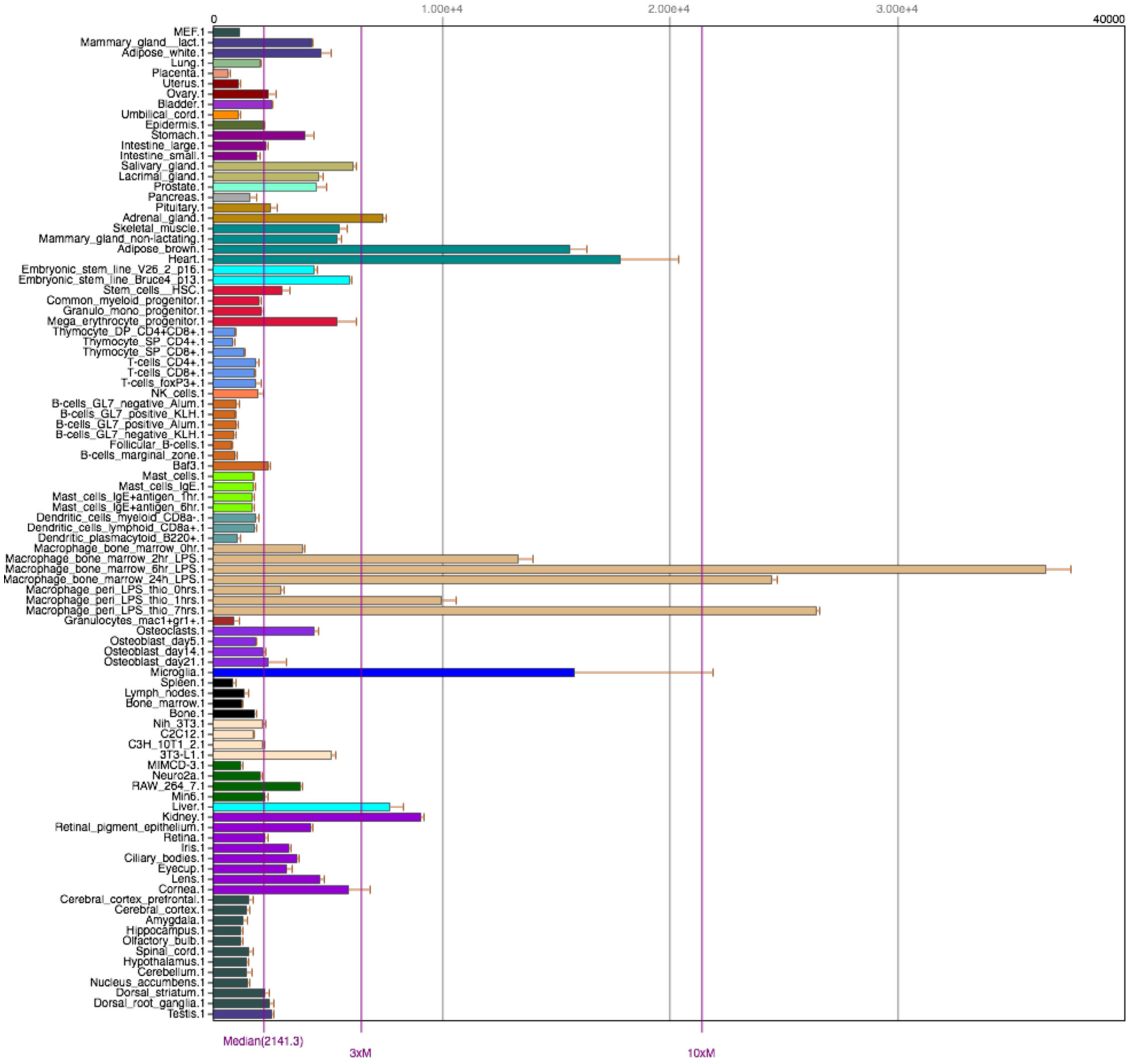

Figure S1. Tissue-specific mRNA expression of SOD2. Shown is mRNA expression of SOD2 in different mouse tissues. Data are generated from BioGPS (www.biogps.org).

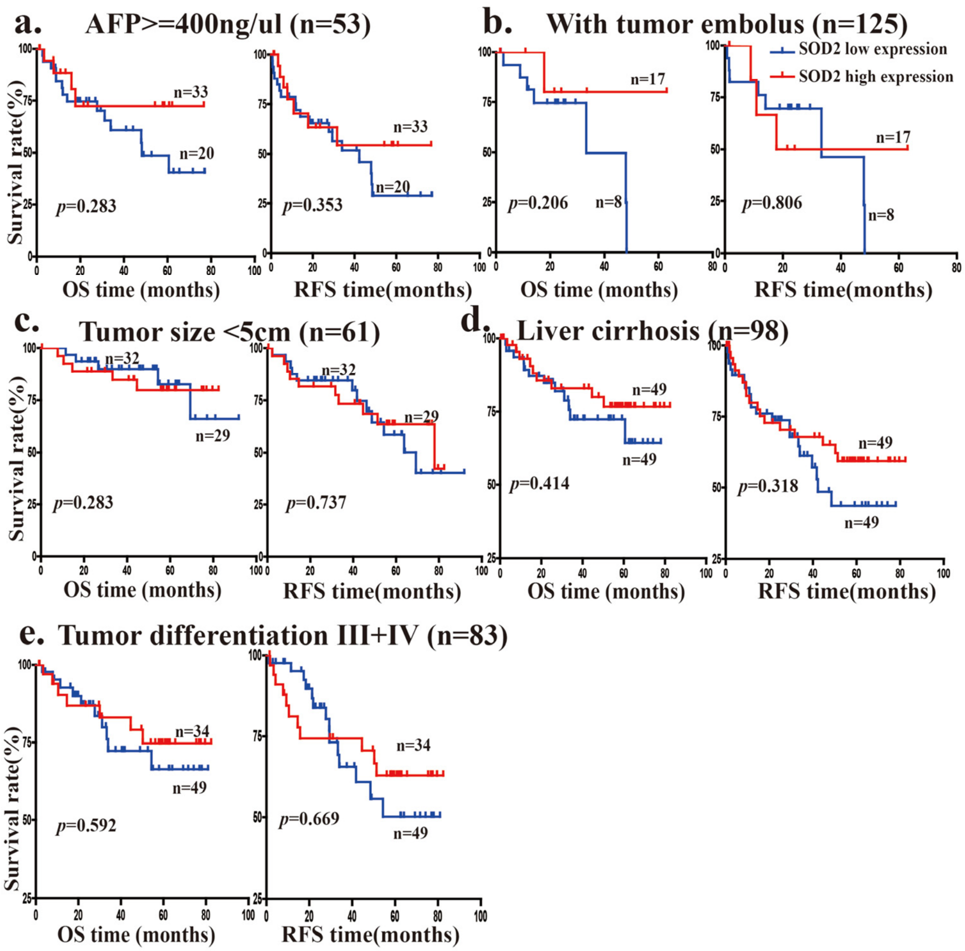

Figure S2. Overall survival (OS) and relapse-free survival (RFS) in advanced HCC patients with different SOD2 protein expression. The OS and RFS of 160 HCC patients with SOD2 high expression and low expression are analyzed in (**a**) patients with high AFP level (> 400 ng/µl), (**b**) patients without tumor embolus, (**c**) patients with tumor size < 5cm, (**d**) patients no liver cirrhosis, (**e**) patients with tumor differentiation III+IV.

Table S1. Patients clinicopathological features

Features	Median (range)
**SOD2 expression : IHC score**	180 (10-300)
**Age: years**	49 (23-77)
**AFP: ng/mL**	51.9 (0.91-724631)
**Tumor size: cm**	6 (1-20)
	**No. of cases (%)**
**No. of patients**	160 (100%)
**Tumor Differentiation**	
I	18 (11.3%)
II	65 (40.6%)
III	73 (45.6%)
IV	4 (2.5%)
**TNM Stage**	
I	100 (62.5%)
II	20 (12.5%)
III	40 (25%)
**BCLC Stage**	
0	8 (5.00%)
A	94 (58.8%)
B	29 (18.1%)
C	29 (18.1%)
**Recurrence**	
No	97 (60.6%)
Early (<24 mons)	39 (24.3%)
Late (≥24 mons)	24 (15.1%)
